# Risk Factors for Death from Pandemic (H1N1) 2009, Southern Brazil

**DOI:** 10.3201/eid1708.101233

**Published:** 2011-08

**Authors:** Renata T.C. Yokota, Lacita M. Skalinski, Cristine N. Igansi, Libia R.O. de Souza, Betine P.M. Iser, Priscilleyne O. Reis, Eliana N.C. Barros, Eduardo M. Macário, Marilina A. Bercini, Tani M.S. Ranieri, Wildo N. Araújo

**Affiliations:** Author affiliations: Ministry of Heath, Brasilia, Brazil (R.T.C. Yokota, L.M. Skalinski, C.N. Igansi, L.R.O. de Souza, B.P.M. Iser, P.O. Reis, E.N.C. Barros, E.M. Macário, W.N. Araújo);; Health Secretariat of Rio do Sul State, Porto Alegre, Brazil (M.A. Bercini, T.M.S. Ranieri)

**Keywords:** pandemic (H1N1) 2009, influenza, case–control studies, death, risk factor, viruses, Brazil, dispatch

## Abstract

To identify risk factors for death from pandemic (H1N1) 2009, we obtained data for 157 hospitalized patients with confirmed cases of this disease. Multivariate analysis showed that diabetes and class III obesity were associated with death. These findings helped define priority vaccination groups in Brazil.

In May 2009, pandemic (H1N1) 2009 was identified in Brazil (1). In June 2009, the first confirmed death from this disease was reported in southern Brazil. On July 16, 2009, Brazil declared sustained transmission of pandemic (H1N1) 2009, and the case definition for mandatory notification was limited to suspected influenza cases with fever >38°C, cough, and dyspnea or death, i.e., severe acute respiratory infection (1). During July 19, 2009–January 2, 2010, a total of 44,544 pandemic influenza cases were confirmed and 2,051 deaths were reported in Brazil, corresponding to notification and death rates of 23.3 cases and 1.1 deaths per 100,000 population. In southern Brazil, notification and death rates reached 110 cases and 3.0 deaths per 100,000 population, and a 4.6% case-fatality rate was observed among reported patients (2). To identify risk factors for death caused by pandemic (H1N1) 2009, we analyzed data for patients hospitalized with confirmed pandemic (H1N1) 2009 at the beginning of the pandemic in southern Brazil.

## The Study

This study was conducted in 11 hospitals in 4 cities (Passo Fundo, Caxias do Sul, Santa Maria, and Uruguaiana) in Rio Grande do Sul (population 10,914,128 in 2009), the southernmost state in Brazil (3). At the time of this study, these 4 cities accounted for 52% of reported deaths from pandemic (H1N1) 2009 in this state.

All laboratory-confirmed (real-time reverse transcription PCR–positive) pandemic (H1N1) 2009 case-patients hospitalized in July 2009 who had shortness of breath or radiologic evidence of pneumonia and either died (case-patients) or were discharged (controls) were included. A standardized form was used that included data reported by patients who survived or their families (patients who died and patients <18 years of age) and information from medical chart review.

We analyzed factors associated with death by calculating odds ratios (ORs) and 95% confidence intervals (CIs). Variables with a p value <0.10 calculated by bivariate analysis were included in a multivariate unconditional logistic regression model adjusted for age and sex. All statistical analyses were conducted by using Epi Info for Windows version 3.5.1 (Centers for Disease Control and Prevention, Atlanta, GA, USA). A p value <0.05 was considered significant.

**Figure 1:**
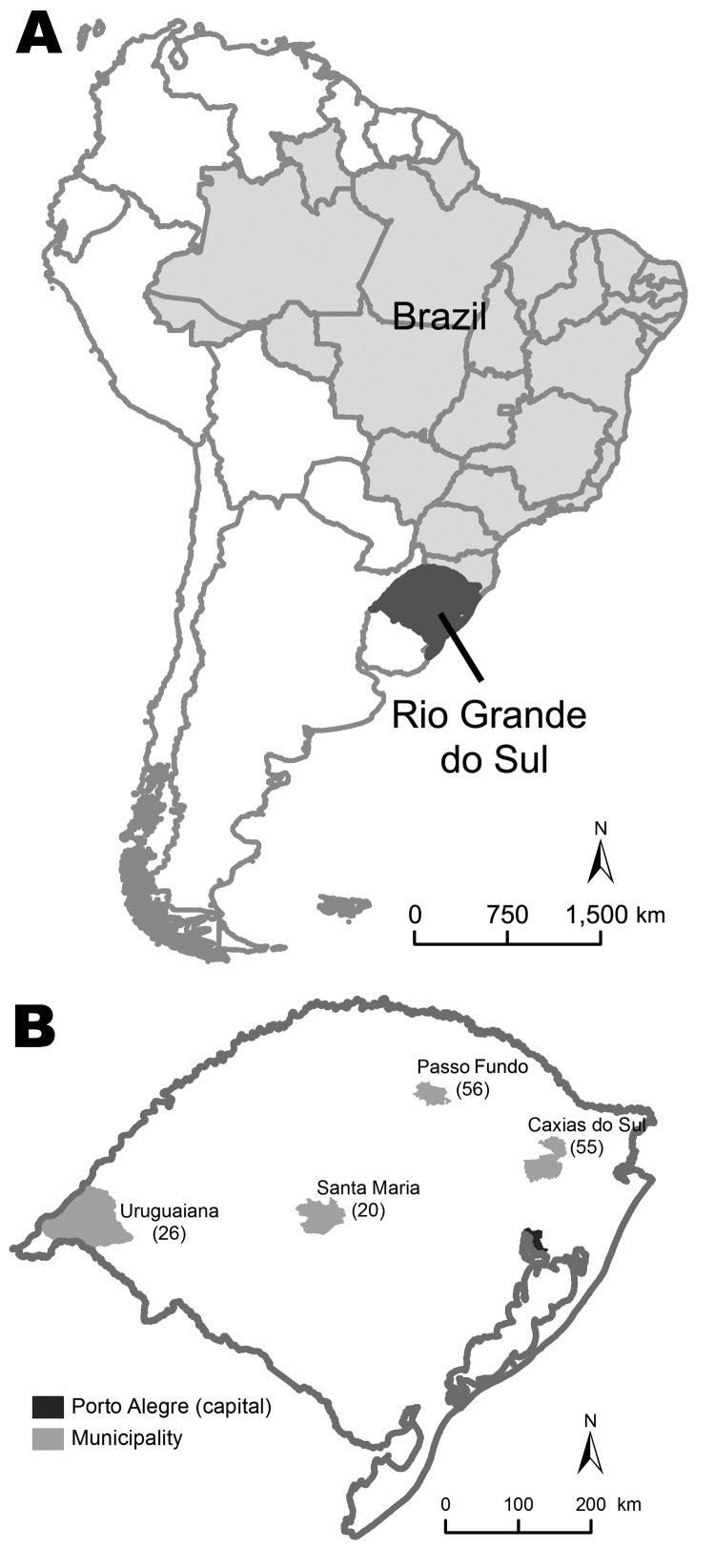
Location of Rio Grande do Sul, Brazil (A) and distribution of 157 patients with pandemic (H1N1) 2009 in 4 cities in this state (B). Values in parentheses are numbers of patients.

**Figure 2:**
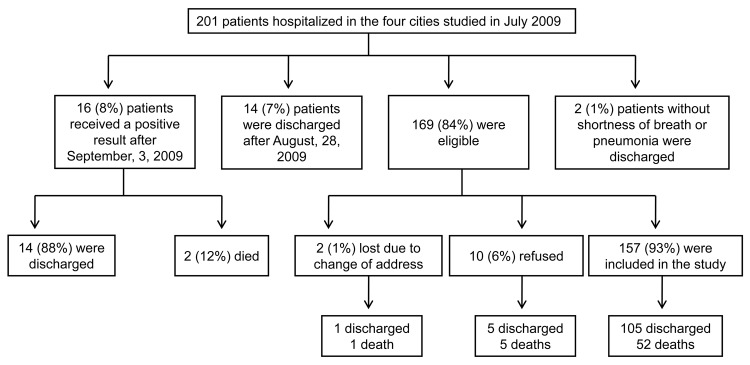
Sample selection process for 201 patients with pandemic (H1N1) 2009, Rio Grande do Sul, Brazil, 2009.

The number of confirmed pandemic (H1N1) 2009 case-patients enrolled in each city is shown in Figure 1. The study included 52 patients who died and 105 who survived (Figure 2). Characteristics and clinical findings of case-patients are shown in Table 1. A total of 136 (87%) of the 157 case-patients sought treatment before hospitalization (median 2 health care visits, range 0–5 visits). Obesity was the most frequent underlying medical condition (38%). Among obese case-patients, 19 (36%) had other risk factors for influenza complications. Diabetes was the most frequent underlying medical condition (21%) in obese case-patients. Thirty-four (49%) of 70 case-patients who did not have risk factors for influenza complications were obese (body mass index >30 kg/m^2^), 6 (9%) had class III obesity (body mass index >40 kg/m^2^), and 20 (29%) had hypertension.

**Table 1:** Characteristics of confirmed pandemic (H1N1) 2009 patients, Rio Grande do Sul, Brazil, 2009*

Characteristic	Value	No. patients
Demographic		
Male sex	78 (50)	157
Age, y	33 (0–73)	157
Family income, US$	678 (0–6,780)	146
Education level, y†	8 (0–19)	129
Residence city different from hospitalization city	46 (29)	157
Smoking habits†		
Current smoker	34 (24)	142
Years exposed to tobacco	14 (1–47)	33
Cigarettes/day	12 (1–60)	33
Pack-years	8 (0–93)	33
Former smoker	19 (13)	142
Years exposed to tobacco	11 (1–54)	18
Cigarettes/day	20 (3–60)	18
Pack-years	6.5 (0–162)	18
Signs and symptoms		
Cough	155 (99)	157
Fever	152 (97)	157
Shortness of breath	152 (97)	157
Myalgia	110 (70)	157
Chills	110 (70)	157
Arthralgia	73 (47)	157
Sore throat	74 (47)	157
Hemoptysis	18 (11)	157
Diarrhea	52 (33)	157
Vomiting	69 (44)	157
Conjunctivitis	9 (6)	157
Headache	56 (36)	157
Seasonal influenza vaccination in the previous year‡	13 (13)	101
Pneumonia vaccination in the previous year‡	5 (5)	101
Health care treatment before hospitalization	136 (87)	157
Risk factor for influenza complication§	87 (55)	157
Diabetes	23 (18)	125
Chronic lung disease	23 (18)	125
Immunosuppression	11 (9)	125
Chronic cardiovascular disease	8 (6)	125
Chronic renal disease	6 (5)	125
Pregnancy trimester¶	15 (25)	59
Second	5 (33)	15
Third	10 (67)	15
Age <5 y	16 (10)	157
Age >60 y	7 (5)	157
Obesity#	53 (38)	138
Class III obesity**	10 (7)	138
Hospitalization		
Admitted to intensive care unit	80 (51)	157
Mechanical ventilation	61 (39)	157
Invasive procedures	69 (44)	157
Clinical complications	54 (34)	157

*Values are no. (%) or median (range). †Children <8 years of age were excluded from the denominator. ‡Influenza and pneumonia vaccination were checked on the vaccination card. §Obesity was not included. ¶Percentage of pregnancy among women of reproductive age (15–49 years) was included. #Body mass index (BMI) data were available for 138 patients. Obesity in adults was BMI >30 kg/m^2^; in children and adolescents <19 years of age, obesity was BMI for age >+2 z scores. Pregnant women were excluded. **Class III obesity was BMI >40 kg/m^2^. Patients <19 years of age and pregnant women were excluded.

Taking medication was reported by 107 (68%) case-patients, but none received oseltamivir before hospitalization. Hospitalization occurred a median of 5 days (range 0–15 days) after symptom onset. Most case-patients (94%) received antimicrobial drugs during hospitalization, and most (81%) began antimicrobial drug therapy on the day of hospitalization. Steroids were administered to 83 (53%) case-patients a median of 1 day (range 0–11 days) after admission.

Three deaths occurred during the first 24 hours of hospitalization. The case-fatality rate was higher among patients admitted to the intensive care unit (47 [59%] of 80 died). No difference was observed between patients who died and those who survived for median number of days between symptom onset and hospitalization (case-patients 6 days, range 0–6 days; controls 5 days, range 0–15 days; p = 0.25) or initiation of oseltamivir treatment (case-patients 6 days, range 1–16 days; controls 5 days, range 0–19 days; p = 0.10). After we adjusted for age and sex, diabetes (OR 4.4, 95% CI 1.5–12.8) and class III obesity (OR 6.2, 95% CI 1.3–29.2) were independently associated with death from pandemic (H1N1) 2009. No association was found between oseltamivir treatment within 48 hours of symptom onset and death (Table 2).

**Table 2:** Characteristics of hospitalized pandemic (H1N1) 2009 case-patients, Rio Grande do Sul, Brazil, 2009*

Characteristic	Outcome, no. (%) case-patients			Unadjusted			Adjusted†	
Died	Survived	OR (95% CI)	p value	OR (95% CI)	p value
Demographic								
Male sex	30 (58)	48 (46)		1.6 (0.8–3.2)	0.16		NC	NC
Current smoker	15 (30)	19 (21)		1.6 (0.7–3.6)	0.21		NC	NC
Former smoker	4 (8)	15 (18)		0.4 (0.1–1.3)	0.13		NC	NC
Underlying medical condition‡	30 (58)	57 (54)		1.1 (0.6–2.2)	0.69		NC	NC
Diabetes	14 (27)	9 (9)		3.9 (1.6–9.8)	0.01		4.4 (1.5–12.8)	<0.01
Chronic lung disease	9 (19)	14 (18)		1.1 (0.4–2.7)	0.87		NC	NC
Immunosuppression	2 (4)	9 (12)		0.3 (0.1–1.7)	0.14		NC	NC
Chronic cardiovascular disease	5 (11)	3 (4)		3.0 (0.7–13.1)	0.13		NC	NC
Chronic renal disease	1 (2)	5 (6)		0.3 (0.1–2.8)	0.27		NC	NC
Pregnancy§	5 (28)	10 (24)		1.2 (0.3–4.2)	0.51		NC	NC
Age <5 y	2 (4)	14 (13)		0.3 (0.1–1.2)	0.06		NC	NC
Age >60 y	4 (8)	3 (3)		2.8 (0.6–13.2)	0.17		NC	NC
Class III obesity¶	26 (57)	27 (29)		5.3 (1.3–21.7)	<0.01		6.2 (1.3–29.2)	0.02
Oseltamivir treatment	25 (48)	64 (61)		0.6 (0.3– 1.2)	0.12		NC	NC
Oseltamivir <48 h after symptom onset	2 (12)	12 (19)		0.4 (0.1–1.8)	0.18		NC	NC
Steroid treatment	32 (71)	51 (57)		0.5 (0.3–1.2)	0.12		NC	NC
Antimicrobial drug treatment	99 (94)	49 (94)		1.0 (0.2–4.1)	0.99		NC	NC

*OR, odds ratio; CI, confidence interval; NC, not calculated. †Adjusted for sex and age group (reference age 0–5 years). ‡Obesity excluded. §Women of reproductive age included (15–49 years). ¶Class III obesity was a body mass index >40 kg/m^2^. Patients <19 years of age and pregnant women were excluded.

## Conclusions

This study confirmed findings from other countries suggesting that at the beginning of the epidemic, pandemic (H1N1) 2009 virus showed a pattern similar to that in the Northern Hemisphere. Consequently, vaccine recommendations in Brazil were made on the basis of epidemiology of pandemic (H1N1) 2009 in Brazil and other countries.

Identification of diabetes and class III obesity as independent risk factors for death caused by pandemic (H1N1) 2009 among hospitalized patients in Brazil was also consistent with findings from other regions (4–8). Prevalence of obesity ranged from 26% to 74% in critically ill pandemic (H1N1) 2009 patients worldwide (5). Diabetes is also considered a risk factor for seasonal influenza complications in nonelderly persons (9). Class III obesity might increase illness and death from influenza because it impedes pulmonary function and contributes to extended mechanical ventilation and hospitalization for these patients compared with nonobese patients (10). Also, class III obesity is frequently associated with other underlying illnesses, such as cardiovascular diseases and diabetes (5).

Diabetes and obesity were overrepresented among case-patients in this study compared with the general population of Rio Grande do Sul. A telephone survey conducted in Porto Alegre (capital of Rio Grande do Sul) found a 14.3% prevalence of self-reported obesity and 6.2% prevalence of self-reported diabetes in 2009 (11). Although we found a low frequency (8%) of class III obesity among patients who died, this frequency was 12.5× the estimate prevalence of class III obesity among adults in Brazil in 2003 (0.64%) (12).

Our study had several limitations. Data were collected retrospectively (median 54 days, range 1–93 days after symptom onset) and by proxy interview for case-patients who died and pediatric patients and were therefore subject to recall bias. Data for analysis, including underlying illnesses and patient weight and height, were not systematically recorded in medical charts. Therefore, these data could not be used to validate questionnaire responses. Furthermore, hospitalized case-patients from whom nasopharyngeal aspirates or swab samples were not obtained were excluded from the study. Thus, the sample analyzed might not be representative of all hospitalized case-patients with severe pandemic (H1N1) 2009 during the study. However, demographic characteristics of study patients were similar to those of reported hospitalized case-patients with suspected pandemic (H1N1) 2009. Conclusions from small case series are limited, and results from this study should be considered in the context of studies in different populations. Quality of hospital care is likely to have a major role in survival rates but is difficult to compare between settings.

To reduce incidence of illness and death, the Brazilian Ministry of Health obtained 110 million doses of monovalent pandemic (H1N1) vaccine for distribution in the first 3 months of 2010. Persons with chronic medical conditions, including diabetes and obesity, received priority for vaccination on the basis of international recommendations (13,14) and those of the Brazilian Ministry of Health (15). In 2010 in Brazil, >89 million persons were vaccinated against pandemic (H1N1) 2009.

**Our study characterized hospitalized case-patients in southern Brazil at the beginning of the pandemic. In addition, we confirmed that class III obesity and diabetes were independent risk factors for death in hospitalized case-patients with pandemic (H1N1) 2009, reinforcing the need for obtaining body mass index data for suspected case-patients during hospitalization. Furthermore, our results contributed to identification of priority groups for pandemic (H1N1) 2009 vaccination in Brazil.**
